# Remarkable Case of Obesity in a Child

**Published:** 1855-07

**Authors:** W. S. Cochran

**Affiliations:** Darlington, Beaver Co., Pa.


### *Remarkable Case of Obesity in a Child.*

By W. S. Cochran, M.D.

Editor of the Medical Examiner:

The subject of this notice residing within a few miles of the village of Darlington, Beaver Co., Pa., is a boy, not three years old till July. The child's mother, together with some others of the family, showed a disposition to corpulency during the first year of their lives, but as soon as they began to walk they became reduced to an ordinary size; hence, in some degree, it might be said to be hereditary in this case. There was nothing remarkable about this child at birth, nor did it show a disposition to "grow fat" till about the seventh month, since which time he has been the wonder not only of the immediate neighborhood, but has attracted visitors from a considerable distance.

The following measurements were taken with the tape applied to the skin, so that they exhibit the *naked fact*:—Measured around the chest 36 inches; abdomen 40; upper part of arm 10; wrist 7; upper part of thigh 26; ankle 12; height 3 feet; weight 98 lbs. He has gained six pounds during the last seven weeks. His head is proportioned to the rest of his body.

With a view to prevent this excessive adipose secretion, he has been confined to a light diet, never using meat, rarely milk, bread and coffee being the articles chiefly relied upon. His intellectual development is fair, about what we would expect from a child of his age who had been considerably among strangers. It is hardly necessary to say that he enjoys the best of health. The case is exceeded by a few on record, yet it is certainly an astonishing example of polysarcia, and one which—if he lives and increase proportionately—will rival the celebrated Lambert.

*Darlington, Beaver Co., Pa.*

